# A Silicon Carbide Wireless Temperature Sensing System for High Temperature Applications

**DOI:** 10.3390/s130201884

**Published:** 2013-02-01

**Authors:** Jie Yang

**Affiliations:** 1 Northeastern University, No. 11, Lane 3, Wenhua Road, Heping District, Shenyang 110819, Liaoning, China; E-Mail: yangjie@ise.neu.edu.cn; Tel.: +86-24-8369-1655; Fax: +86-24-2389-3138; 2 Arkansas Power Electronics International, Inc., 535 W. Research Center Blvd., Fayetteville, AR 72701, USA

**Keywords:** silicon carbide, high temperature, wireless, temperature sensing, thermocouple, gas turbine

## Abstract

In this article, an extreme environment-capable temperature sensing system based on state-of-art silicon carbide (SiC) wireless electronics is presented. In conjunction with a Pt-Pb thermocouple, the SiC wireless sensor suite is operable at 450 °C while under centrifugal load greater than 1,000 g. This SiC wireless temperature sensing system is designed to be non-intrusively embedded inside the gas turbine generators, acquiring the temperature information of critical components such as turbine blades, and wirelessly transmitting the information to the receiver located outside the turbine engine. A prototype system was developed and verified up to 450 °C through high temperature lab testing. The combination of the extreme temperature SiC wireless telemetry technology and integrated harsh environment sensors will allow for condition-based in-situ maintenance of power generators and aircraft turbines in field operation, and can be applied in many other industries requiring extreme environment monitoring and maintenance.

## 1. Introduction

Temperature sensors have a wide array of applications, from household electric appliances to industrial process controls. Many other physical measurements, such as pressure, flow, *etc.*, often times depend on the ambient temperature and require simultaneous temperature measurement to improve their accuracy. There are many different types of commercially available temperature sensors [1,2], among them the most popular temperature sensors include thermocouples [3–5], resistive temperature detectors (RTD) [6,7], thermistors [8,9] and semiconductor temperature sensors [10–12]. Non-contact temperature measurement can be achieved using infra-red thermometers or pyrometers [13].

Although temperature sensing has been well established for decades, acquiring accurate temperature information in certain harsh environments, especially at high temperature, is still a very challenging task. For example, current sensing technology allows for rudimentary internal monitoring of turbine engines. However, extremely sophisticated, complex, and heavy data retrieval systems and wiring harnesses, that are realistic only in a test lab, are still required to route the sensing data out of turbine engines. On rotating components inside the engine such as turbine blades, harsh conditions can include temperatures up to 1,700 °C and centrifugal forces above 10,000 g, as well as being corrosive and debris-laden. Health monitoring of these components involve drilling the turbine disk and rotor in order to route wires for sensing elements, and then extracting the data via a slip ring or conventional telemetry package [14]. Drilling engine components results in significant reductions in component life, as well as causing a delay in engine build schedules. To date, such sensing systems have been used principally during turbine development and performance evaluation, but not in field operation.

There are several sensing technologies that could be used at high temperature, such as thermocouples, optical sensors, surface acoustic wave (SAW) sensors and RF coupling sensors. Platinum-based thermocouples (types R, B and S) are very stable at high temperature up to 2,000 °C [15]. However, the sensitivity of high temperature thermocouple is generally low and could be buried by the induced common-mode noise. In optical sensors the characteristics of optical signal such as intensity, polarization, phase or spectrum are modulated by the temperature stimulus. Various optical sensors for high temperature applications have been developed, including remote pyrometers (radiation thermometers) [16–18], thermal expansion thermometers [19,20], fluorescence thermometers [21–24], and thermometers based on optical scatterings [25–28]. Optical sensors are favorable for their small size, light weight, and resistance to electromagnetic interference (EMI). However, the temperature limitation of the optical fiber affects the applicability of the optical sensors in certain hostile environments. SAW sensors and RF coupling sensors for high temperature sensing have also been studied recently, and some SAW sensors have already been demonstrated in gas turbines up to 1,100 °C [29–36]. SAW sensors utilize the phase velocity variation of surface acoustic wave on piezoelectric substrate at different temperatures, while the RF coupling sensors detects the frequency change of a LC resonator whose capacitance (*i.e.*, the dielectric constant of material) is correlated to the temperature. Both SAW sensors and RF coupling sensors have the advantages of being wireless and batteryless. However, SAW sensors are very sensitive to the variation of environmental, geometric and material properties that can usually be seen in harsh environments, while RF coupling sensors are inherently prone to EMI, in addition to their limited high temperature capability, below 300 °C.

**Figure 1.**
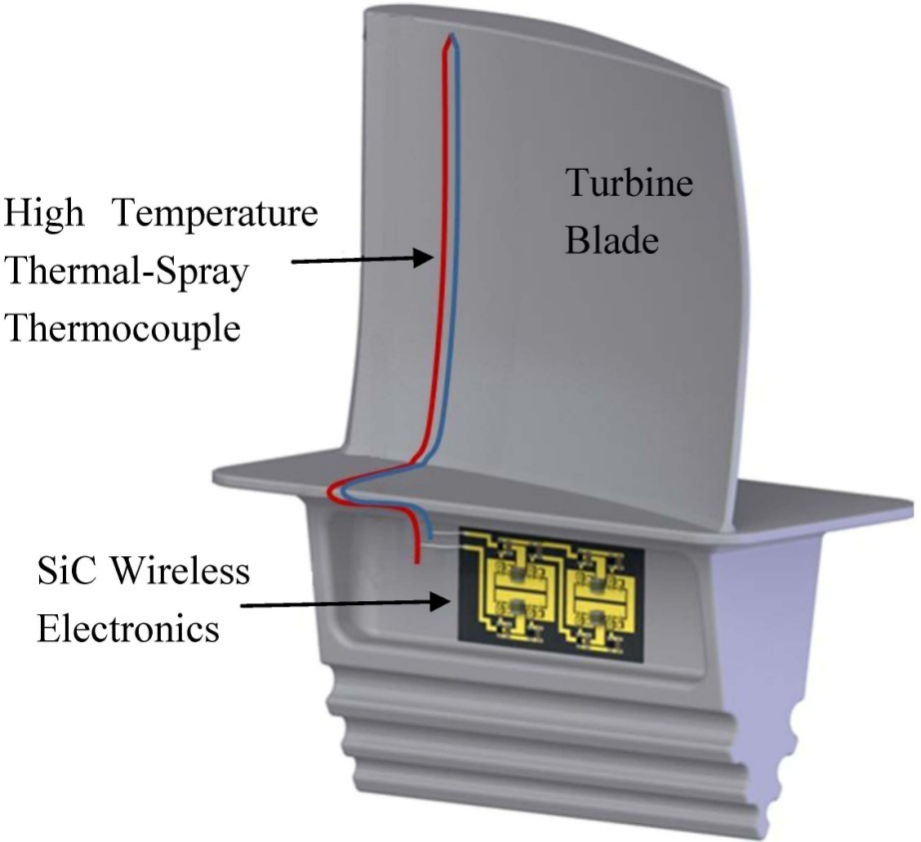
Conceptual configuration of proposed wireless temperature sensing system for turbine blade health monitoring.

A promising solution to the aforementioned problem is to integrate an extreme environment-capable wireless transmitter with a high temperature thermocouple, and embed the entire sensor-transmitter system onto the critical components of turbine engine. As such, the information acquired by the sensor can be wirelessly transmitted to a receiver outside the turbine unit. This kind of wireless telemetry system is traditionally implemented based on silicon technology [37] with an upper temperature limit of 200 °C (with proper thermal insulation). Recently, the start-of-art high temperature SOI (HTSOI) technology has also been adopted in the sensor industry, which raises the temperature limit of the integrated electronics to 350 °C. Unfortunately, even the HTSOI technology cannot fulfill the temperature requirement for the sensing system inside the turbine engine, for which the most advanced wide band-gap (band-gap greater than 1.7 eV) technologies, such as silicon darbide (SiC), gallium nitride (GaN) and aluminum nitride (AlN), whose theoretical temperature limit is up to 600 °C [38], have to be considered. In this article, a SiC-based, highly miniaturized wireless temperature sensing system capable of operating at an extreme temperature of 450 °C is introduced. A conceptual diagram of this proposed sensing system in a turbine blade health monitoring application is shown in Figure 1. It consists of a thermal-spray thermocouple sprayed directly onto the surface of the turbine blade, and a SiC based high temperature wireless transmitter integrated in the basement of the turbine blade. Since the thermocouple and the wireless transmitter are closely located, without a long lead wire in-between, the signal attenuation and the induced common-mode noise will be minimized. The SiC wireless transmitter is exposed to the high temperatures for extend periods of time, during which the availability of passive and active electrical devices are greatly limited and with much degraded performances. Therefore, innovative circuit designs are required to achieve the electrical functionalities at such extreme temperature. In addition, novel packaging technologies, including substrate fabrication, die attachment, wire bonds, *etc.*, are also essential to meet the temperature and mechanical requirements.

In Section 2, the system level design of the proposed SiC wireless transmitter and the core SiC transistor is introduced. Electric designs of major functional circuit blocks of the SiC wireless transmitter are detailed in Section 3. Several important packaging issues under harsh environments are discussed in Section 4. High temperature test results are summarized in Section 5, followed by concluding remarks in Section 6.

## 2. System Design of SiC Wireless Transmitter

**Figure 2.**
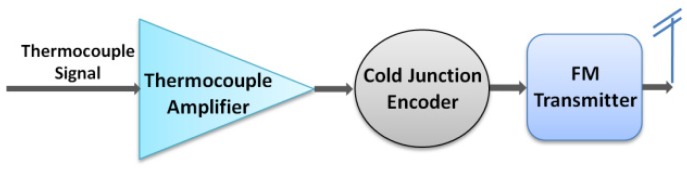
System block diagram of the proposed wireless temperature sensing system.

Figure 2 shows the system block diagram of the wireless transmitter for thermocouples. It consists of three major components: a thermocouple amplifier, a cold junction encoder and a frequency modulated (FM) transmitter. The full-scale signal output from the thermocouple is very low (0–10 mV typical) and mixed with surrounding electrical noise. Therefore, the signal must first be conditioned and amplified to a proper level for future signal processing. Also, the output of the thermocouple is a DC-like signal representing the slowly varying temperature. It has to be converted into AC format before being transmitted. In addition, the thermocouple measures the temperature difference between the hot end and cold junction where the transmitter is located. In order to obtain the absolute temperature at the hot end (which is the measurement of interest), both the thermocouple signal and the local temperature at the cold junction must be available. The cold junction encoder senses the local temperature and embeds it into the thermocouple signal, while simultaneously converting the signal into a square-wave AC signal. Once the thermocouple signal is processed, it modulates a RF carrier through a FM modulator. Compared various analog modulation schemes such as amplitude modulation (AM) and phase modulation (PM), FM is the best candidate for its improved noise immunity (compared to AM) and lower complexity implementation (compared to PM) [39,40]. The modulated RF signal is then amplified to an appropriate power level and radiated through an antenna.

**Figure 3.**
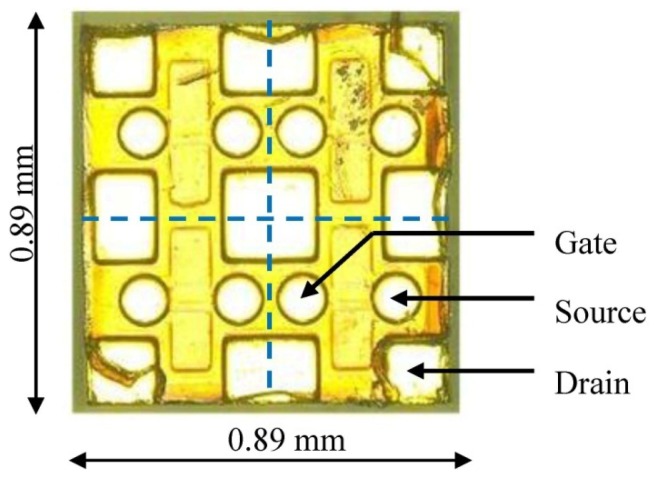
A custom-built SiC VJFET specifically designed for low power RF applications.

Since the majority of research efforts for wide band-gap devices focus on power electronics operating at relatively low temperature below 250 °C, they are not suitable for the proposed wireless electronics. Therefore, the core transistor of the proposed SiC wireless transmitter is a custom-built SiC n-channel vertical junction field-effect transistor (JFET) especially optimized for high temperature, high frequency and low power consumption operation. As the traditional packaging approach is not applicable at such desired high temperature, this SiC JFET is in the bare die form as shown in Figure 3. Each die contains four JFETs sharing a common drain terminal.

**Figure 4.**
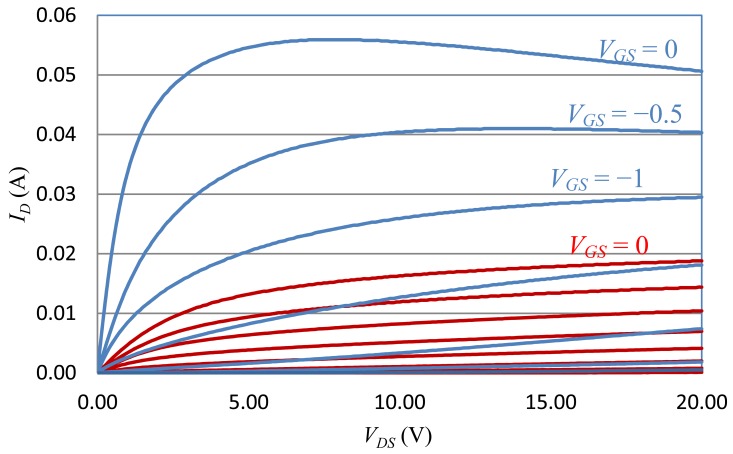
The on-state curves of the SiC vertical JFET at 25 °C (blue) and 525 °C (red).

This custom-built SiC VJFET transistor was fully characterized from room temperature up to 525 °C using a Signatone RF Probe Station and Sony/Tektronix 372 Curve Tracer. The on-state curves of the JFET at 25 °C and 525 °C are depicted in Figure 4 in blue and red, respectively. Furthermore, an empirical full temperature range hyperbolic SPICE model was also built to facilitate the circuit design and simulation [41]. The hyperbolic Spice model of SiC VJFET can be expressed as:

(1) $$I_{D}=\beta {\left(V_{GS}-V_{TO}\right)}^{2}(1+\lambda V_{DS})\tanh(\alpha V_{DS})$$

where *I_D_* is drain current; *V_GS_* is gate to source voltage; *V_TO_* is threshold voltage, and *α*, *β* and *λ* are temperature-dependent parameters being optimized from the measured data. For this particular SiC JFET, these parameters are given by:

(2) $$\left\{ \begin{array}{l} \alpha =0.19236\\ \beta =0.01164\\ \lambda =0.0011e^{0.0042268T} \end{array}\right.$$

From Figure 4, it can be clearly seen that some important characteristics of the transistor, such as transconductance, change drastically with temperature elevation. Besides the SiC JFET, the characteristics of other high temperature active and passive components such as diodes, resistors, capacitors and also depend on the ambient temperature. Therefore, special attention has to be paid to this fact and temperature compensation techniques are required in high temperature circuit design.

## 3. High Temperature Circuit Design of SiC Wireless Transmitter

The biggest challenge of circuit design comes from the limitation of devices. Since the SiC n-channel JFET is the only transistor satisfying all of operational requirements of the system, and very few other active and passive devices are operational in the designed temperature range, creative circuit designs are essential to achieve proper functionality of the system. As shown in Figure 2, the entire wireless transmitter is divided into several functional circuit blocks. In this section, circuit design of the key functional blocks will be detailed.

### 3.1. High Temperature Differential Amplifier

**Figure 5.**
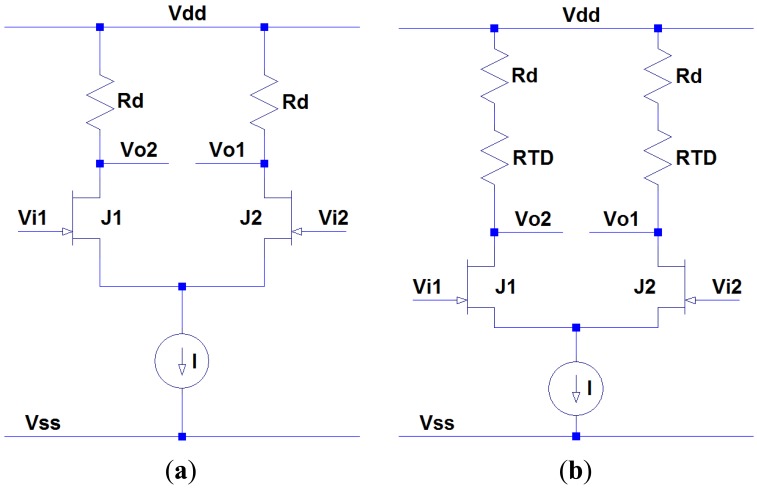
(**a**) Classic resistive load JFET differential amplifier. (**b**) Modified JFET differential amplifier with additional RTD elements for temperature compensation.

The first circuit block is the thermocouple amplifier. Since the signal output from the thermocouple is typically very low and mixed with surrounding electrical noise, a JFET-based differential amplifier with a high common-mode rejection ratio (CMRR) is desired to filter out common mode noise coupled in the thermocouple signal, while simultaneously amplifying the signal strength to a proper voltage level. Figure 5(a) shows a classic resistive load differential amplifier consisting of a pair of matched JFETs, load resistors and a current source. In ideal case, the differential gain of this differential amplifier is given by:

(3) $$A_{d}=\frac{V_{od}}{V_{id}}=\frac{V_{o2}-V_{o1}}{V_{i2}-V_{i1}}=g_{m}R_{d}$$

where *g_m_* is the transconductance of the JFETs, which is proportional to the differential gain. According to Figure 4, the transconductance of the SiC JFET reduces by approximately 60% when ambient temperature raises to 450 °C, resulting in either insufficient amplification at high temperature or over-amplification at low temperature. Fortunately the differential gain is also proportional to the load resistance. Therefore, RTD elements are introduced in this differential amplifier circuitry to compensate the gain loss at high temperature, as shown in Figure 5(b). For this circuit schematic, the modified differential gain can be written as:

(4) $$A_{d}=\frac{V_{od}}{V_{id}}=\frac{V_{o2}-V_{o1}}{V_{i2}-V_{i1}}=g_{m}(R_{d}+\text{RTD})$$

**Figure 6.**
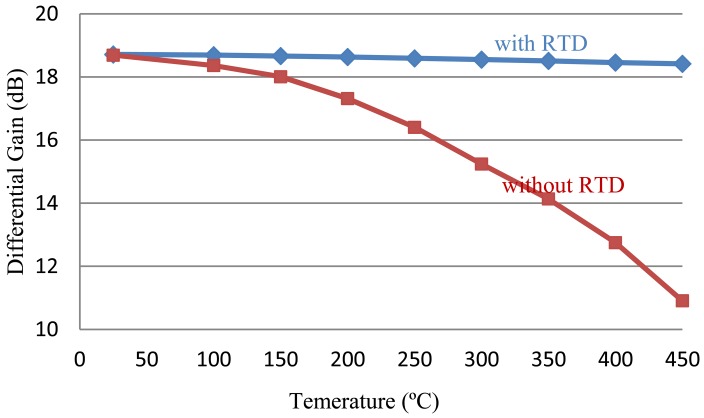
Differential gain of the SiC JFET differential amplifier with and without RTD temperature compensation.

The resistance value of RTD element is a monotone increasing function of temperature, so it can effectively compensate the gain loss caused by the reduction of transconductance. By carefully adjusting the resistance ratio between *R_d_* and RTD, the variation of differential gain can be minimized when temperature change occurs. Platinum RTD element is an excellent candidate for this purpose, due to its large temperature coefficient, very linear temperature response and better stability at high temperature. The differential gains with and without RTD elements are depicted in Figure 6. The differential gain reduces by only 4% (0.3 dB) when RTDs are utilized, compared to the 59% (7.8 dB) differential gain reduction without RTDs. Therefore, the high temperature performance of differential amplifier is improved significantly.

**Figure 7.**
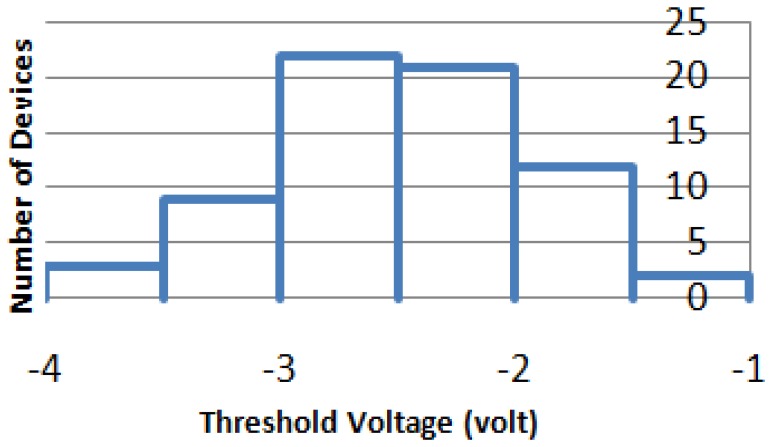
Distribution of SiC VJFTs with respect to threshold voltage.

Another important figure of merit for differential amplifier is CMRR. The key of obtaining high CMRR is to closely match the symmetric devices (including JFETs and load resistors) in differential pairs [42]. However, due to the issue of SiC wafer uniformity and fabrication imperfection, the operation characteristics (especially the threshold voltage) of SiC JFETs are widely spread, as shown in Figure 7. As such, each SiC VJEFT must be individually characterized and a strict device matching process has to be involved in system development.

### 3.2. High Temperature Astable Multivibrator

**Figure 8.**
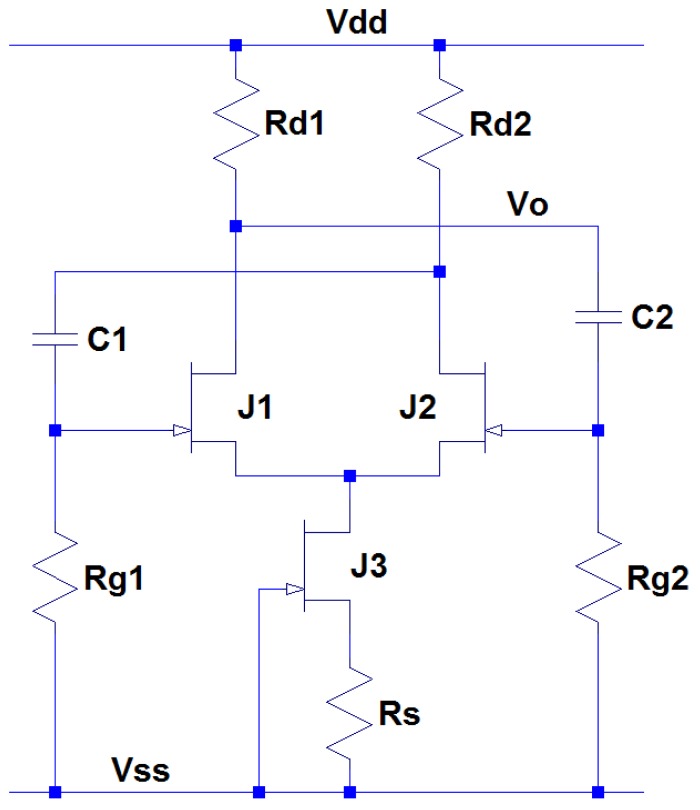
Circuit schematics of SiC JFET astable multivibrator whose frequency is temperature-dependent.

The next circuit block is the cold junction encoder, which converts the DC-like thermocouple signal into an AC format and embeds the temperature of the circuit board into the signal. This configuration allows both hot end and cold junction temperature information to be transmitted together by a single channel transmitter so that the transmitter design can be greatly simplified. The cold junction encoder is implemented using a JFET switching circuit driven by a square waveform, generated from an astable multivibrator (relaxation oscillator). The circuit schematic of this astable multivibrator is depicted in Figure 8. The oscillating frequency of this circuitry is given by:

(5) $$f=\frac{1}{\ln2\times (R_{g1}\times C_{1}+R_{g2}\times C_{2})}$$

For the special case of 50% duty cycle, (*i.e.*, transistors J_1_ and J_2_ have the same on and off time) *R_g1_* = *R_g2_* and *C_1_* = *C_2_*, Equation can be simplified as:

(6) $$f=\frac{1}{2\ln2\times R_{g}\times C}$$

**Figure 9.**
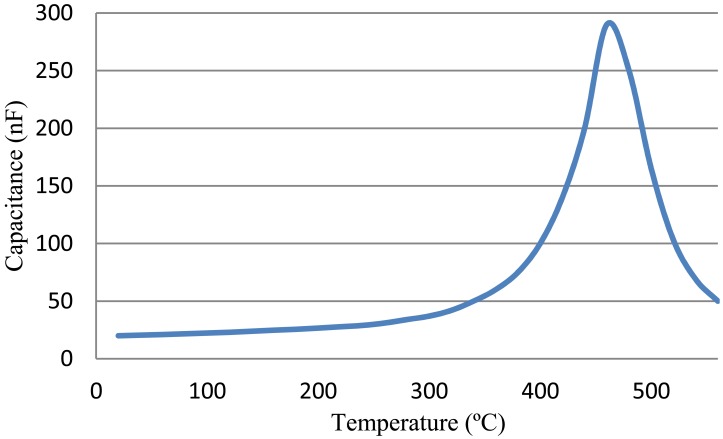
Capacitance value of the high temperature X7R type capacitor.

It can be seen that the oscillating frequency of this astable multivibrator is inversely proportional to the resistance *R_g_* and capacitance *C*. The key elements of this multivibrator are the two X7R-type capacitors whose capacitance is temperature dependent. Figure 9 shows the measured capacitance of this capacitor over temperature, with a peak at approximately 460 °C. It can be seen that the capacitance of this capacitor is a monotonously increasing function of temperature from 20 °C to 450 °C; thus, a square wave with decreasing frequency is expected when the temperature increases. In real circuit, this X7R-type capacitor is connected to with a NPO-type capacitor (whose capacitance is temperature stable) in serial to smooth out the frequency shif over temperature.

**Figure 10.**
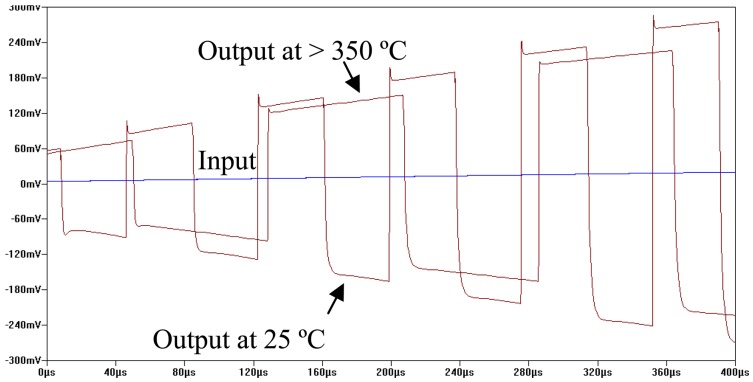
Simulation result of signal conditioning circuitry of thermocouple SiC wireless transmitter.

In order to fully utilize the differential gain of the differential amplifier, a three-way switching circuit is implemented to take both outputs from the differential amplifier and switch back and forth from these differential outputs. This yields a square wave output signal whose amplitude carries the relative temperature information between hot-end and cold junction and whose frequency carries the absolute temperature information of the cold junction. Therefore, the desired absolute temperature information of hot-end can be calculated by combining amplitude and frequency of the output signal. The simulation result of the signal conditioning circuitry is depicted in Figure 10 when a small linearly increasing DC voltage is fed into the differential amplifier as input signal. It can be seen that the amplitude of output signal is proportional to the amplitude of input signal while the frequency of them are distinguishable at different temperatures.

### 3.3. High Temperature Voltage Controlled Oscillator (VCO)

**Figure 11.**
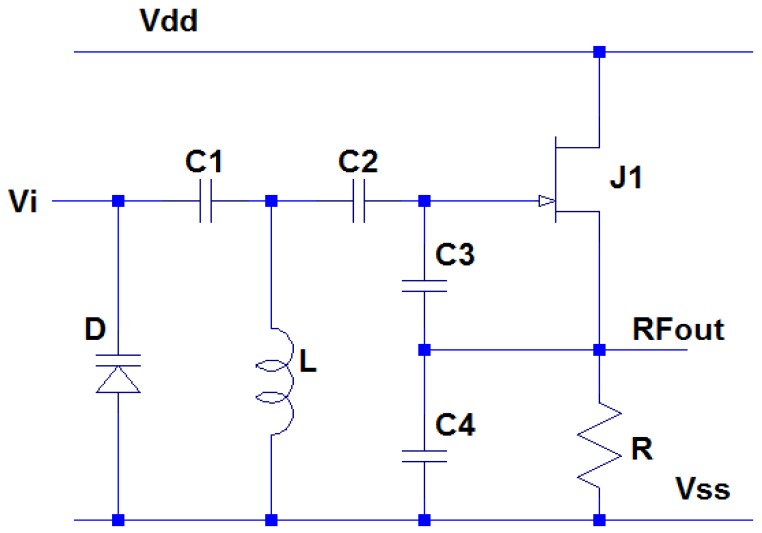
The circuit schematic of high temperature SiC JFET Clapp VCO.

The last circuit block is the FM transmitter. The transmission circuitry consists of a VCO and a RF power stage coupled to an output antenna. The signal from the three-way switch will be used to modulate a RF carrier signal by means of direct frequency modulation. This results in the instantaneous frequency deviation about the center carrier frequency being proportional to the amplitude of the modulating signal, and the rate of deviation equal to the frequency of the modulating signal [43]. A Clapp VCO configuration is utilized and the circuit schematic is depicted in Figure 11.

The frequency of this oscillator is set by the inductor L and the capacitance combination of capacitors *C_1_*, *C_2_*, *C_3_*, *C_4_* and varactor *D* as follows:

(7) $$f=\frac{1}{2\pi }\sqrt{\frac{1}{L}\left(\frac{C_{1}C_{D}}{C_{1}+C_{D}}+\frac{C_{2}C_{3}C_{4}}{C_{2}+C_{3}+C_{4}}\right)}$$

**Figure 12.**
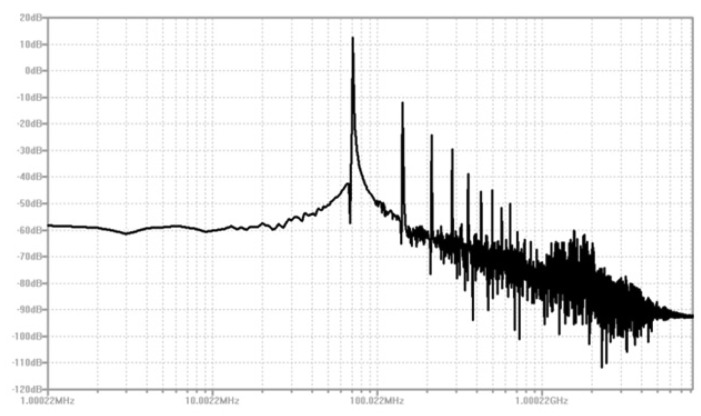
Simulation result of RF oscillator at 450 °C.

When an input signal is applied to the varactor, the voltage variation of the signal will alter the capacitance of varactor and will eventually modulate the RF carrier frequency generated by the Clapp oscillator. The key component of the Clapp oscillator is the inductor *L* with the ability to maintain high quality factor at high temperature, which can be achieved by printing a planar inductor directly onto the ceramic substrate. Figure 12 shows the simulation result of RF oscillator, indicating that a RF carrier at approximately 70 MHz is expected.

Once the carrier signal has been generated and modulated, it is power amplified and delivered to a buffer that serves to isolate the VCO from any external loading, as this can shift the frequency of the oscillator in a significant and unpredictable manner [44]. The buffered signal is radiated wirelessly through a transmitting antenna. The signal is then captured by an FM receiver and demodulated to recover the desired temperature information. The maximum data rate of the signal depends on the bandwidth of the FM system. Currently an industrial highly-sensitive FM receiver is used, whose bandwidth is limited to 75 kHz.

## 4. High Temperature Packaging Consideration

Electronic packaging is an extremely challenging task for the proposed wireless sensing system due to the simultaneous high temperature and high g-force requirements for turbine blade health monitoring applications. Critical packaging aspects, including substrate materials, metallization, die attachment, wire bonding process, system packaging and materials interactions have to be carefully considered and novel packaging techniques need to be developed to accomplish this task.

The temperature limit of common printed circuit board (PCB) materials designed for high temperature operation, such as polyimide, does not have long-term survivability at temperatures over 250 °C. Alternatively, ceramic-based substrates, including alumina and low temperature co-fire ceramic (LTCC), are promising candidates for high temperature circuit substrate materials. Alumina substrate has the advantage of simple circuit fabrication for fast prototyping. However, LTCC is more desirable for its multilayer capability, which results in highly miniaturized packaging. In addition, cavity structures can be made in LTCC substrates to place the discrete circuit components (JFETs, capacitors, resistors, *etc.*) that will provide lateral mechanical support to these components to overcome the sheer force caused by the centrifugal load. Therefore, LTCC is utilized in the prototype fabrication of this SiC wireless temperature sensing system.

Wire bonding is another one of the major sources of failure. Cratering under the bond pad, heel fracture, joint lift due to contamination, voiding and the formation of brittle intermetallics, all leading to premature bond failure. Many of these mechanisms are further accelerated at high temperatures. Some conventional wire bonds have been shown to survive temperatures up to 600 °C [45], but the addition of extreme centrifugal forces simultaneously with elevated temperatures can cause severe reliability problems. The direction of wire bond also significantly affects the reliability of wire bond connection. When the wire bonds are perpendicular to the g-load, it is more easily to be laid over and touch the substrate, which causes the connection failure. Another important aspect of packaging is reliable die attachment. High temperature solder was tested and resulted in lateral die fractures after the attachment process or thermal cycling due to the excessive residual stress. Therefore, a solid state attachment method that reduces system stress by allowing for processing temperatures near the final operating temperature was developed. The entire transmitter is packaged in a gold plated Kovar package for maximum mechanical ruggedness.

**Figure 13.**
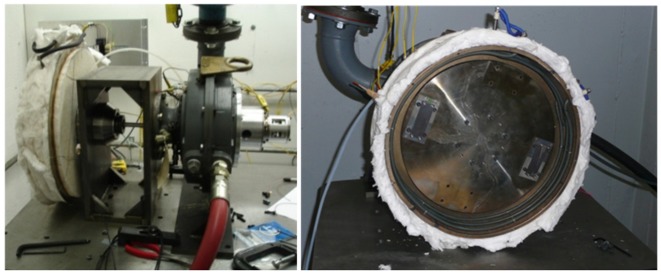
Spin test rig developed by Aerodyn Engineering, Inc. for high temperature and high g-load testing. **Left**: entire test rig. **Right**: inside the chamber.

In addition, high temperature spin testing is employed to test all of the packaging processes and methods. The spin test rig developed by Aerodyn Engineering, Inc. (Indianapolis, IN, USA) for high temperature and high g-load testing is illustrated in Figure 13. This rig is comprised of an air turbine which includes a spin arbor for mounting the electronics, a heater to bring the electronics up to temperature, and slip rings to run signals in and out of the electronics during testing. All of the various aspects of the electronic system are characterized before and after spin testing to determine the effects the extreme forces impose on the electronics. In addition, electrical functionality can also be tested during the spin test.

## 5. Prototype and High Temperature Test Results

**Figure 14.**
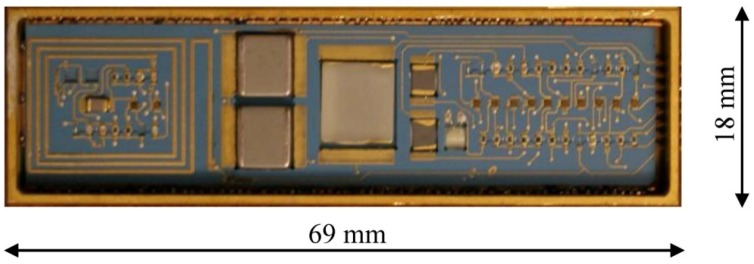
A prototype electronic system packaged with high temperature and high g-load capable die attaches and wire bonds.

Once the electric design and packaging research were finalized and validated, a SiC wireless transmitter prototype combining both high temperature SiC electronics technology and harsh environment packaging technology was fabricated, as shown in Figure 14.

**Figure 15.**
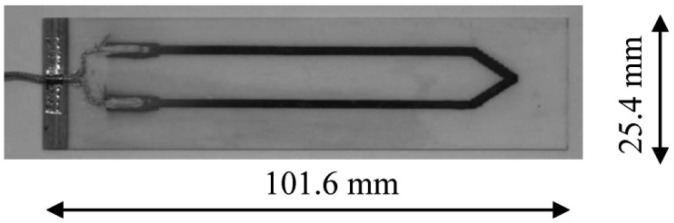
Direct-Write thermocouple test coupon provided by MesoScribe, Inc.

This prototype was tested in the lab environment using hotplates, and direct-write thermocouples thermally sprayed on alumina substrates provided by MesoScribe, Inc. (St. James, NY, USA), as shown in Figure 15, were also integrated into the high temperature test setup. During the testing, the thermocouple was heated by a hotplate to emulate the temperature difference between the neck of the turbine blade where the wireless transmitter will be located and the tip of the blade, where the thermocouple sensing end will be located. The prototype was placed on another hot plate to emulate the high temperature environment the wireless transmitter exposes to. Another standard K-type thermocouple was utilized as reference for system calibration. While the power and ground were delivered through connecting probes, the RF signal was picked up from a distance of 150 mm via a whip antenna connected to Tektronix RSA3303B real-time spectrum analyzer. The received signal was then demodulated directly by the spectrum analyzer to recover the modulating waveform. During the testing, a Tektronix TDS5034B oscilloscope is also utilized to record the output of signal conditioning stage.

**Figure 16.**
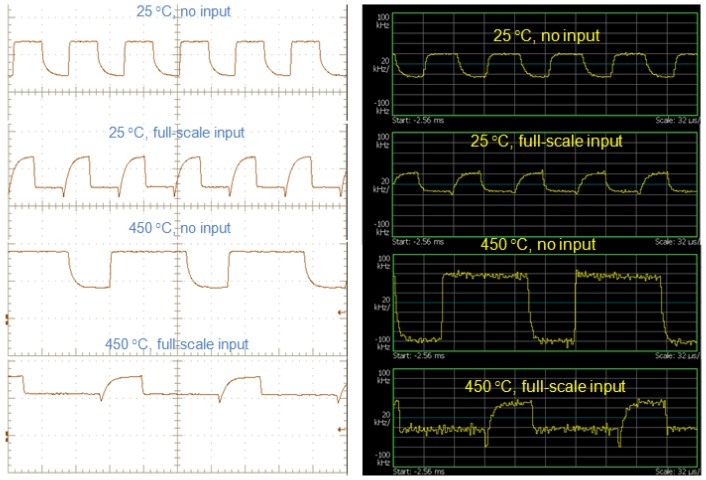
Comparison between output waveform from signal conditioning stage and the demodulated waveform from RF carrier at different temperatures.

The test results were recorded from room temperature to 450 °C. At each measurement temperature, the outputs of the circuit were measured under two scenarios: with and without thermocouple input. For the case without thermocouple input, the thermocouple input is shorted. Therefore, the input voltage to the signal conditioning block (*i.e.*, voltages across the two gate terminals of differential pair) is equal to 0 V. For the case with thermocouple input, the thermocouple is plugged into the input of the signal conditioning stage, resulting in a small DC input signal. At each temperature of interest, the output of signal conditioning stage and the received RF signal were recorded. The received RF signal was then demodulated using 500 kHz span around the peak spectrum by real-time spectrum analyzer. Figure 16 shows the comparison between the output waveform of signal conditioning stage at 25 °C and 450 °C recorded by digital oscilloscope (left column) and the waveform demodulated from RF carrier (right column). Several arguments can be made from examination of the measured results:

1. At each temperature, the waveforms from signal conditioning stage and the demodulated waveforms resemble each other. It clearly indicates that the desired temperature data are transmitted wirelessly to the receiver and successfully recovered through the demodulation process over the entire temperature range.
2. Output waveform has certain magnitude even though the input signal is not presented. This is mainly due to device mismatching. Even though the JFETs are carefully paired, different JFETs may have different temperature coefficients, which make it practically impossible to match devices over the entire temperature range. However, this initial offset will not affect the temperature measurement as long as it is fully characterized.
3. The polarity of the waveform switched when the from zero thermocouple input to full-scale input. The system is tuned this way for RF bandwidth consideration. In field operation, a commercial high-sensitive RF demodulator will be used, which has limited bandwidth of 150 KHz for each channel. If the full-scale output signal has the same polarity as the initial offset caused by the device mismatching, a large portion of the bandwidth will be wasted by the initial offset, which in turn will reduce the temperature resolution of the sensing system.
4. The demodulated waveform becomes noisy at 450 °C. The main reason for that is the signal strength of RF carrier decreases at high temperature due to the degraded performance of SiC JFETs and other passive components. In addition, the thermal noise of circuit components also contributes to the noisy output as it is no longer negligible at such extreme temperature.

**Figure 17.**
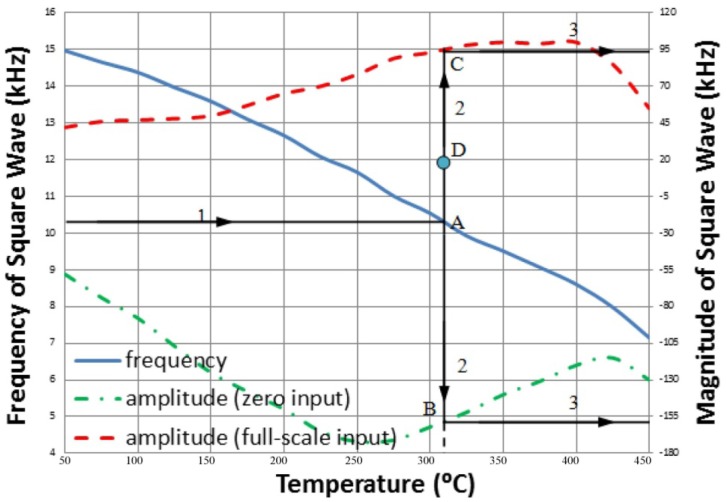
Characteristic curves of the wireless temperature sensing system prototype.

The characterization curves of the thermocouple wireless transmitter prototype were summarized in Figure 17. The blue solid line represents the frequency of the demodulated square waveform, while the green dash-dotted line and red dashed line are the amplitude of the demodulated waveform when thermocouple input is zero and full-scale, respectively. For a given demodulated waveform, the first step is to determine the local temperature based on the frequency of the waveform. Then the full scale amplitude of output at such temperature can be found. Based on the full scale amplitude and the amplitude of received waveform, the relative temperature (*i.e.*, the thermocouple signal) can be calculated. Finally, the addition of relative temperature and the local temperature gives the absolute temperature of interest. As such, the entire sensing system is able to recover the absolute temperature information being measured by the thermocouple from the received RF signal, in real-time fashion.

## 6. Conclusions

In this research work, a miniaturized extreme environment thermocouple wireless sensing system is developed through successful demonstration of a fully functional prototype from 25 °C to 450 °C. In addition to the electrical design of the SiC high temperature wireless transmitter circuitry, much effort was also allocated to the development of extreme environment packaging technologies to ensure reliable circuit operation under high temperature and mechanical loads. Another important aspect of this wireless sensing technology is the power source. Currently an induced power deliver system, including a set of transmitting coils mounted on the stator of the turbine and a receiving coil co-located with the wireless transmitter, is being developed to continuously power the wireless sensing system. A power conditioning circuitry consisting of an AC-DC converter and a voltage regulator will also be integrated into the SiC wireless transmitter to provide a stable voltage source.

The extreme temperature SiC electronics free the sensor from the requirements of extremely difficult and costly wire harness and cabling, and yield a highly integrated and miniaturized sensing system, which, along with its wireless functionality, will make it possible to be embedded into the turbine engines without major engine structure modifications. The wireless temperature sensing system show great potential in providing *in situ* health monitoring in the extreme environments encountered within turbine engines and other similar applications.
